# Changes in the ability to correctly identify schizophrenia and depression: results from general population surveys in Germany over 30 years

**DOI:** 10.1007/s00127-024-02660-y

**Published:** 2024-04-07

**Authors:** Elise Grohmann, Amani Al-Addous, Christian Sander, Ezgi Dogan-Sander, Eva Baumann, Matthias C. Angermeyer, Georg Schomerus

**Affiliations:** 1 Department of Psychiatry and Psychotherapy, University of Leipzig Medical Centre, Leipzig, Germany; 2 Department of Journalism and Communication Research, Hannover University of Music, Drama, and Media, Hannover, Germany; 3 Centre for Public Mental Health, Gösing am Wagram, Austria

**Keywords:** Mental health literacy, Trend survey, Stigma, Labelling, Major depression, Schizophrenia

## Abstract

**Purpose:**

This study aims to examine time trends in the ability to correctly identify schizophrenia and major depression within the German general population from 1990 to 2020, as an indicator of changing mental health literacy (MHL). Additionally, we investigated shifts in the use of stigmatizing language.

**Methods:**

Our analysis is based on four waves of representative population surveys in Germany in 1990/1993 (West Germany: *N* = 2044, East Germany: *N* = 1563), 2001 (*N* = 5025), 2011 (*N* = 2455), and 2020 (*N* = 3042) using identical methodology. Respondents were presented with an unlabelled case vignette describing a person who exhibited symptoms of either schizophrenia or major depression. Participants were then asked to name the problem described in the vignette using an open-ended question.

**Results:**

From 1990/1993 to 2020, correct identification of schizophrenia increased from 18% to 34% and from 27% to 46% for major depression. However, derogatory labels remained constant throughout all survey waves, particularly for schizophrenia (19% in 1990/1993 and 18% in 2020). For depression, more trivializing and potentially devaluing statements were recorded.

**Conclusion:**

Despite the increasing use of psychiatric terminology among the general population, the persistence of derogatory labels suggests that improved MHL, reflected in higher recognition rates, may not automatically translate into a reduction in stigmatizing language. With depression, a normalization and trivialization of a severe illness could pose new challenges to people with major depression. Dedicated efforts to combat the stigma of severe mental illness are still needed.

**Supplementary Information:**

The online version contains supplementary material available at 10.1007/s00127-024-02660-y.

## Introduction

Mental health literacy (MHL) describes the broadest possible knowledge of the causes, symptoms, treatment strategies, and prevention measures of a psychiatric diagnosis [1, 2]. This knowledge is a main precondition for the ability to correctly identify mental illness as such in real life [3]. The accurate identification and labelling of mental illnesses by members of a society may serve as prerequisites for early help-seeking and effective treatment, potentially leading to improved prognoses [4–7]. Since Jorm first introduced the concept of MHL in 1997 [1], various population groups worldwide have been studied to assess their MHL regarding different mental disorders. Overall, a steady increase in MHL has been observed, especially in industrialized countries [8–14]. MHL is typically assessed using questionnaires that elicit knowledge of causes, symptoms, or treatment strategies by asking respondents to rate or agree/disagree with different pre-defined options [15]. For example, the evaluation of the English Time-To-Change campaign showed that a growing share of the population agreed that terms like “schizophrenia” or “depression” denominate a mental illness [16]. Overall, the ability to correctly recognize and label an acute mental health problem and distinguish it from everyday problems is in fact an important aspect of MHL. However, in real life, particularly when encountering an acute mental health crisis, people are not presented with questionnaires but with complex social situations and have to rely on their active knowledge and internalized mental models of different types of human behaviour [17]. Only very few population studies use unlabelled case vignettes and record responses using open-ended questions, thus assessing active, unprompted MHL. For instance, a study conducted in South Australia found an increase in the proportion of individuals correctly identifying depression and acknowledging their personal experiences with depression over 6 years between 1998 and 2004 [12]. In a large representative study by our research group, respondents were inquired about their association with “schizophrenia” and then asked open-ended questions on causes and treatment recommendations. While those respondents who felt confident enough to answer the questions largely favoured professional medical care and psychosocial or biological causes, about half of the respondents were unable to come up with any potential cause or treatment recommendation [18].

In this paper, we report data from a four-wave, repeated cross-sectional long-term study on beliefs and attitudes about mental illness spanning three decades from 1990 to 2020. Surveys in all four waves used an identical, unlabelled vignette of someone showing symptoms of either schizophrenia or depression and an identically worded, open-ended question about the nature of the problem described. Hence, we have the unique opportunity to describe the long-term trajectory of MHL in the general population in Germany regarding their ability to recognize and label acute and severe mental illness.

The aim of our study is thus to determine the proportion of respondents in each wave since 1990 that were able to identify the problem described in the vignette as a mental disorder or were even using a broadly correct label. Given the open-ended nature of our questions, other aspects of labelling mental health issues can also be investigated. The language used by survey participants to describe the condition in the case vignette may reveal potentially stigmatizing attitudes when people use derogatory labels. Given that a lack of knowledge about mental disorders is described as a contributory factor to stigmatization processes [5, 19], we hypothesized that improved abilities to correctly recognize a mental illness could lead to reduced use of derogatory language. Moreover, the premise that precise recognition of mental conditions requires substantial engagement with the subject indicates that such an approach could collectively promote understanding and acceptance, dismantle stereotypes, and amplify empathy [20, 21].

## Methods

### Surveys

In 1990/1993, 2001, 2011, and 2020, we conducted four waves of a representative population survey among German-speaking individuals aged 18 or older living in private households. The original survey was conducted in West Germany before reunification in 1990 (*N* = 2044, response rate 70%), followed by a survey in the former East German regions in 1993 (*N* = 1563, response rate 71.2%), thus, both surveys are combined for our analysis to cover both West and East Germany in the early 1990s. All subsequent surveys were conducted nationwide (2001: *N* = 5025, response rate 65.1%; 2011: *N* = 2455, response rate 64%; 2020: *N* = 3042, response rate 57%). The methodology of the surveys was identical: The samples were selected using 3-stage random sampling (for more details, see supplementary material), and fully structured interviews were conducted face-to-face by trained interviewers. In 2020, due to the COVID-19 pandemic, there was an additional option for participants to fill out the questionnaire on their own while the interviewer waited outside. This option was chosen by 15.3% of participants in that year.

In 1990 and 1993, sampling and data collection were carried out by GETAS Hamburg, while in the subsequent years, the fieldwork was conducted by USUMA Berlin. Both companies are renowned and specialize in market and opinion research. At the beginning of the interviews, participants were informed verbally about the background and purpose of the study, as well as the voluntary nature of participation and their right to withdraw. Every participant provided written consent. The participation was not remunerated. Our study was approved by the review board of Greifswald University Medical Centre (BB 195/18).

### Samples

The sociodemographic characteristics of our samples across the survey waves are presented in Table 1, complemented by the socio-structural data on the German population during the corresponding years. Overall, the samples can be considered approximately representative of the German population at the respective time points of the surveys.

**Table 1:** Comparison of the sociodemographic characteristics of our samples with those of the general German population during the respective survey years

	Survey 1990/1993	Total population in 1990 ^A^	Survey 2001	Total population in 2000 ^A^	Survey 2011	Total population in 2010 ^A^	Survey 2020	Total population in 2019 ^C^
Gender								
Male	46.2	48.5	43.8	48.3	44.9	48.6	47.2	49.3
Female	53.8	51.5	56.2	51.7	53.6	51.4	52.4	50.7
Diverse							0.4	
Missed					1.5			
Age, years								
18–25	11.5	12.3	11.7	9.8	8.4	11.3	10.4	10.9
26–45	38.7	38.0	38.4	37.8	29.7	31.9	32.2	30.4
46–60	24.1	24.2	23.9	23.3	28.5	26.9	28.5	27.3
+ 60	25.3	25.5	25.2	29.1	33.2	29.9	28.9	31.4
Missed			0.8		0.2			
Educational attainment^B^								
Still student	2.3	0.4	2.8	0.2	1.0	1.0	1.1	0.2
No schooling completed	4.4	2.5	3.8	2.1	3.3	4.0	1.6	4.4
8/9 years of schooling	40.7	55.8	43.7	49.1	38.5	38.5	28.2	33.0
10 years of schooling	34.5	25.8	32.2	27.5	39.0	29.3	41.1	26.3
12 years of schooling	18.2	15.5	17.4	21.1	18.2	27.1	28.0	36.1

Percentages of sample/population

^A^Data from the federal statistical office of Germany

^B^Only persons  ≥ 20 years; population data for younger persons is not available

^C^Data from the federal statistical office of Germany [© Statistisches Bundesamt (Destatis), 2021 | Stand: 05.01.2021 / 11:52:42]

### Interviews

At the beginning of the interviews, respondents were presented with a diagnostically unlabelled female or male case description of schizophrenia or major depression based on DSM-III-R criteria. Before their use in the initial survey in 1990, each vignette underwent diagnostic confirmation by five experts in psychopathology to ensure an accurate diagnosis. The respective wording of the vignettes can be found in the supplementary material. Vignette gender and type were randomly assigned, except in 1993 (surveys conducted only in East Germany) and in 2001, when only the male case vignette was used. The distribution of schizophrenia and depression vignettes was evenly split (Table S1 in the supplementary material). Immediately after the presentation of the vignette, participants were asked the open-ended question: “How would you describe the condition that this person is in? What do you think this person has?” Multiple answers were possible and recorded verbatim. In the course of the interview, the participants were only asked closed-ended questions, the results of which have been published elsewhere [22, 23].

### Coding of open-ended responses

Starting in 1990/1993, all spontaneously expressed answers regarding the description and labelling of the symptoms described in the vignette were coded according to an inductively developed category system, grouped into 13 main categories (Table S2 in the supplementary material). While the 13 main categories remained unchanged during all subsequent waves, sub-categories were amended in each survey if new labels emerged. For example, within the main category “medical language”, the sub-category “burnout” was introduced in 2011, having been used by 10.2% of respondents presented with the depression vignette. Re-analysing the 2001 data then showed that this new category was also applicable to 0.3% of answers in the previous survey [24].

In general, a code was assigned when the content of a category was mentioned either literally or paraphrased in the response of a study participant. During this process, codes were also assigned for the absence or implausibility of responses, ensuring the absence of missing data in our statistical analyses. Since many participants provided several responses (for example, “she is distressed; she has depression”), each response was coded separately, leading to up to six codes per respondent. Among the participants, the distribution of codes varied, with the majority (7563 participants) receiving one code, followed by 4372 participants receiving two codes, 1607 participants receiving three codes, 469 participants receiving four codes, 95 participants receiving five codes, and 13 participants receiving six codes.

For this study, three thematic groups of categories were selected (for more details, see Table S3 in the supplementary material). These groups comprised sub-categories of different main categories and were established following the aim of our study. Specifically, we focus on the following groups of categories: (1) 'Use of medical language', [including and distinguishing use of a 'correct diagnosis' (1a), for instant *schizophrenia, paranoid, or psychosis* for the schizophrenia vignette; 'incorrect diagnosis' (1b), such as *anxiety disorder*, or *depression* if used for the schizophrenia vignette; and cases when respondents cited both 'correct and incorrect diagnoses' (1c)]. For the depression vignette, among other categories, we classified 'burnout' as an incorrect diagnosis. However, since it is widely perceived as a correct technical term for states of depression in the general population [25], we conducted an additional analysis of the use of 'burnout for depression' (1d).

We identified 'derogatory labels' that discredit and mark the people described in the vignette as deviating from the social norm negatively, such as *crazy*, *insane*, *malingerer*, or *idiot* (2a). For those labels that were not clearly devaluing but could be perceived as either trivializing or devaluing, we introduced a further category, 'labels that may be perceived as derogatory or trivializing' such as *should have a holiday*, *has no responsibilities*, or *should sleep in* (2b).

### Statistical analysis

We conducted comparative exploratory statistical analyses of spontaneous responses to the depression and schizophrenia vignettes, examining the frequency distribution of categorical variables across all four waves of data collection. Binary logistic regression models were employed to calculate the effect of time (time points 1990/1993 and 2020) as the primary predictor (independent variable) on the manifestation of the following categories (dependent variables): the use of medical language and correct and incorrect diagnoses. Gender, age, and education level of the respondents, as well as the gender of the vignette, were controlled as potential confounding factors. Additionally, we assessed education level and vignette gender as potential moderators of the effects of time (interaction terms). Cases lacking gender or age data underwent listwise exclusion in the regression analyses. All data analyses were performed using IBM SPSS Statistics 21.

## Results

### Use of medical language

**Fig. 1:**
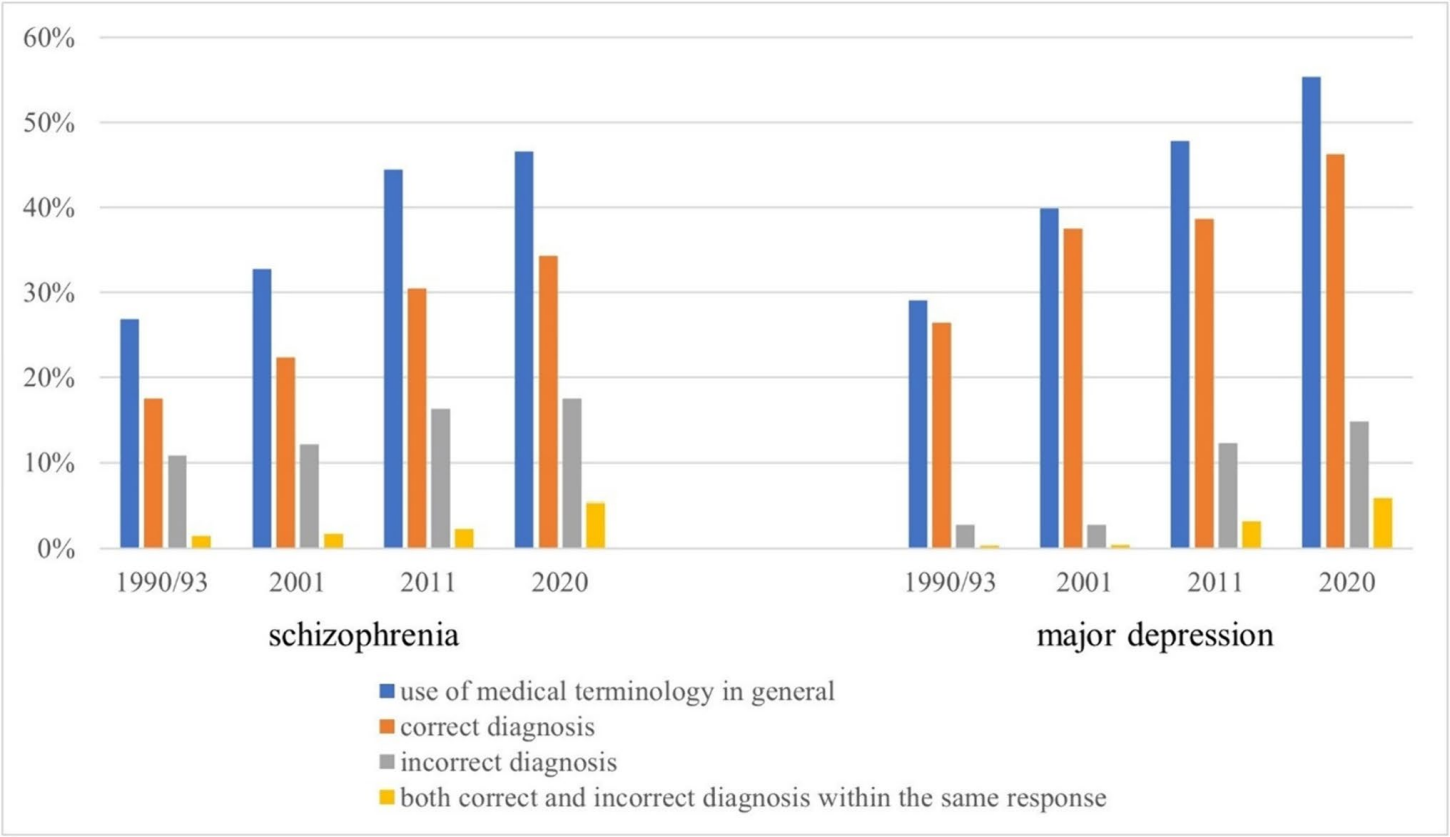
Use of medical language for schizophrenia and depression between 1990/1993 and 2020. Observed numbers (in per cent—Table S4 supplementary material) of respondents acquired in 1990/1993 (schizophrenia–*N* = 2115, depression–*N* = 1492), 2001 (schizophrenia–*N* = 2481, depression–*N* = 2544), 2011 (schizophrenia–*N* = 1235, depression–*N* = 1220), and 2020 (schizophrenia–*N* = 1512, depression–*N* = 1530) in West and East Germany using male and female character vignettes of depression or schizophrenia

Figure 1 shows an increase in the use of medical language in general for both described mental illnesses, schizophrenia and major depression, over the three decades covered by our study.

In 1990/1993, only one out of four respondents (26.9%) used medical language to describe the schizophrenia vignette, while in 2020, 46.6% used medical language (odds ratio (OR) 2.29, 95% CI 1.96–2.67, *p *< 0.001), indicating that, if all other factors were held constant, respondents in 2020 were 2.29 times more likely to use medical language than those in 1990/1993. For the depression vignette, the use of medical language increased even more, from 29.1% in 1990/1993 to 55.4% in 2020, or approximately 26 percentage points (OR 2.97, 95% CI 2.52–3.50, *p *< 0.001).

The development of the use of correct and incorrect medical terms differed for both disorders. For schizophrenia, 17.5% provided a correct diagnosis and 10.8% an incorrect diagnosis in 1990/1993; hence, more than half of all medical terms were incorrect. In 2020, this proportion slightly improved: While in this most recent survey, 34.3% described the schizophrenia vignette with a correct medical term, we found incorrect use of medical language in 17.5% of respondents. Overall, both the use of correct medical language (OR 2.33, 95% CI 1.96–2.78, *p *< 0.001) and the incorrect use of medical language (OR 1.80, 95% CI 1.45–2.22, *p *< 0.001) increased for statements regarding the schizophrenia vignette. Among the incorrect terms, about one-third in both 1990/1993 and 2020 were related to the term “depression/depressive”.

In descriptions of the depression vignette, the use of the correct diagnosis also increased from a higher baseline of 26.5% in 1990/1993 to 46.3% in 2020 (OR 2.35, 95% CI 1.99–2.78, *p* < 0.001). With 2.8%, the percentage of incorrect diagnoses was noticeably lower in 1990/1993 than for the schizophrenia vignette (10.8%). Its increase over the years to about 15% was almost exclusively due to a rise in the use of burnout (burnout to describe depression—2001: 0.4%, 2011: 10.2%, 2020: 11.8%).

Comprehensive statistical data for the analysis can be found in Table S5 of the supplementary materials.

### Derogatory labels

**Fig. 2:**
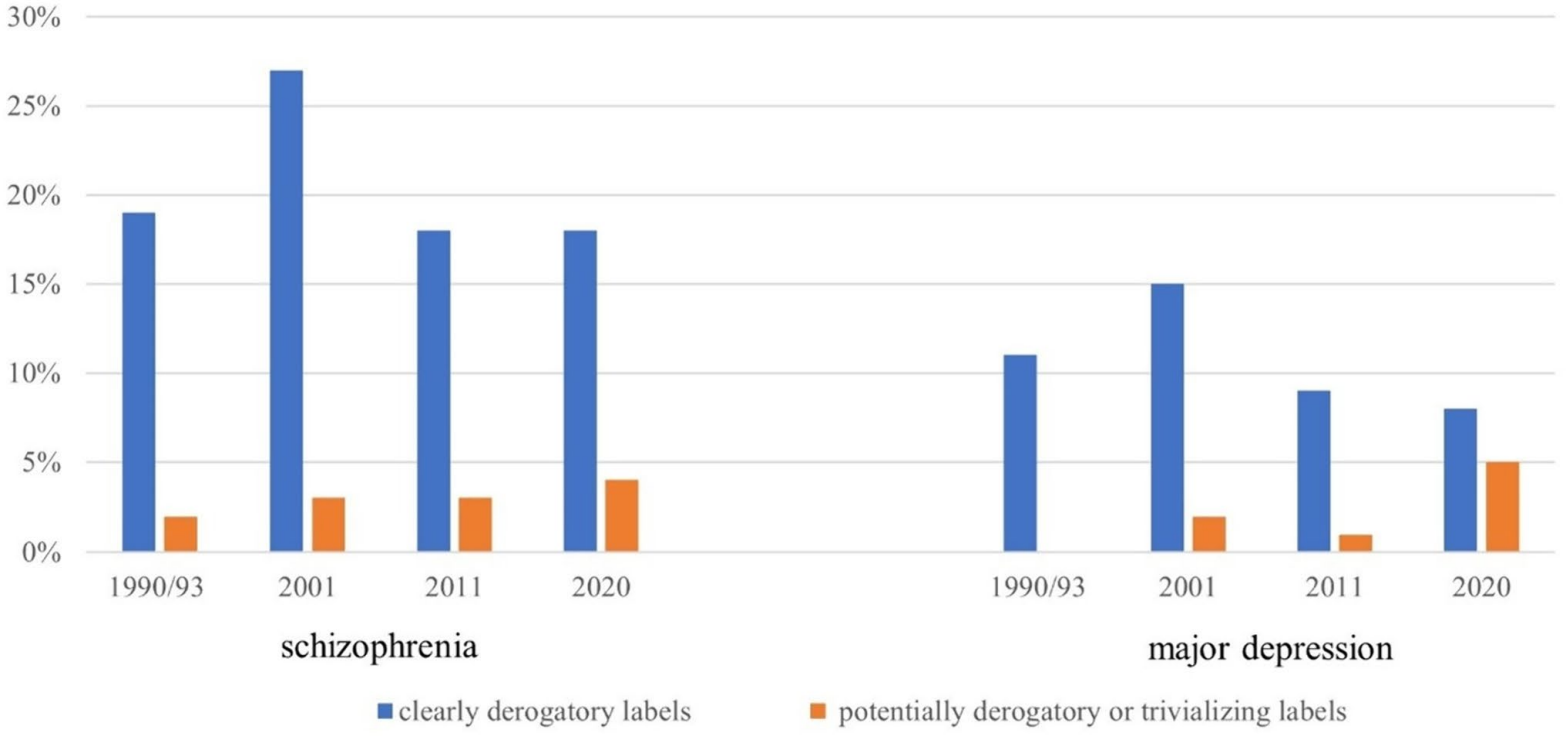
Development of using derogatory labels for schizophrenia and depression between 1990 and 2020. Observed numbers (in per cent—Table S4 supplementary material) of respondents acquired in 1990/1993 (schizophrenia–*N* = 2115, depression–*N* = 1492), 2001 (schizophrenia–*N* = 2481, depression–*N* = 2544), 2011 (schizophrenia–*N* = 1235, depression–*N* = 1220), and 2020 (schizophrenia–*N* = 1512, depression–*N* = 1530) in West and East Germany using male and female character vignettes of depression or schizophrenia

Figure 2 shows the percentages of respondents using clearly derogatory labels and potentially derogatory or trivializing labels from 1990/1993 to 2020.

More terms that are derogatory were used in response to the schizophrenia vignette compared to the depression vignette. For both disorders, derogatory labels peaked in the 2001 survey. For schizophrenia, they remained fairly constant in 1990/1993, 2011, and 2020, while in depression, a shift towards potentially derogatory and trivializing, but not openly stigmatizing labels was observed. While 'clearly derogatory labels' for the depression vignette decreased from 10.7% in 1990 to 7.6% in 2020, the share of 'potentially derogatory or trivializing labels' increased from 0.4% in 1990/1993 to 5.2% in 2020. This shift was far less pronounced in schizophrenia.

## Discussion

Our study shows that the use of medical language to describe a person with symptoms of severe mental illness has increased since 1990, for both schizophrenia and depression. For schizophrenia, the percentage of derogatory labels used by respondents remained overall constant at about 20%. For depression, we observed a slight decline in clearly derogatory labels from 11 to 8%, but an increase in potentially derogatory and trivializing terms.

Before discussing our findings, we need to evaluate the strengths and limitations of our study. Our investigation stands out as the only vignette-based study examining trends over 30 years. The application of the same open-ended question to elicit labels for identical case vignettes throughout this time is unique. However, the long study period also brings about some methodological challenges. For the 1990 interviews, the original answers were not preserved verbatim due to technical restrictions on the amount of data storage at the beginning of the 1990s; hence, we were unable to revise the codes assigned at that time in the case of newly added sub-categories. However, the coding system provided extensive verbatim examples for each code, so we are confident that the categories were applied consistently over the entire period. Further, the combination of the 1990 (West) and 1993 (East) surveys led to an overrepresentation of people living in East Germany for that wave. However, prevalence in categories did not differ meaningfully between East and West German respondents at that time; hence, this procedure should not have had an impact on our findings (for more details, see supplementary material in Table S6).

In 1993 and 2001, only the male version of the vignettes was used, which likely affected the results for those years. In both the earlier and later surveys, we observed slight variations in the frequency distributions of categories based on the presented gender of the vignette, particularly regarding the use of derogatory statements (refer to Table S7 in the supplementary material). However, these differences did not prove to be significant.

We used case vignettes describing someone with severe, untreated mental health symptoms. Thus, developments in attitudes towards milder courses of depression or schizophrenia, or towards people who are already in treatment, are not covered by our study. While focusing on unprompted label assignments for an unlabelled case vignette seems adequate to capture the ability to correctly recognize and label a mental health illness, other aspects of MHL, like knowledge about treatment options or actions that should be taken in a concrete situation, are not covered by our study. Across the survey waves, there was a decreasing response rate, from 70% in 1990 to 57% in 2020. Declining response rates are observed in many social science time-trend studies [26], although the face-to-face interview method still performs better than, for example, telephone surveys in this regard.

Finally, the use of an open-ended question poses a difficulty in quantitative analysis and constrains the statistical possibilities due to their nominal distribution. However, open-ended questions provide an unprompted, undistorted view of the individual's perception of the phenomenon described and thus may serve as a very direct indicator of MHL in the general population. At the same time, the willingness and ability of the interviewees to articulate themselves cannot be dismissed as a potential bias.

Considering those limitations, our results nonetheless indicate an overall increase in the prevalence of psychiatric terms in the general population, with the term “depression” being used more readily than “schizophrenia”. This corresponds to the higher lifetime prevalence of depression, estimated at 15–20% (point prevalence 5–8%) [27–30] compared to schizophrenia, with a lifetime prevalence of 2–4% (point prevalence around 0.4%) [30–32]. It also reflects public awareness and media coverage [33–35]. Depressive disorders are more frequently mentioned and receive generally more favourable and balanced media coverage compared to schizophrenia. A German newspaper analysis found that in 2019, coverage of schizophrenia in print media occurred about half as often as coverage of depression and was often solely concerned with crime and violence, without mentioning illness concepts, treatment modalities, or prognosis. The coverage of depression, in contrast, was much more balanced, with ample reference to treatment [36]. Additionally, an increasing number of celebrities who have publicly acknowledged their own or their close ones' experience of depression has likely contributed to the spread of psychiatric terminology cantered around depression and to a de-tabooing in terms of perceived normalization, getting used to explicitly naming and recognizing depressive symptoms [37, 38].

Several findings of our study illustrate normalization, particularly in dealing with depressive symptoms, and the term “depression”. Approximately one-third of the responses that were categorized as correct labelling used the adjective “depressive.” “Depressive”, however, has already permeated everyday language and is sometimes used synonymously with “down” or “low”, thus blurring the line between medical and everyday language. We also encountered colloquial variations of “depression” like “depri”, which we coded separately.

Our observation that the term “depression/depressive” was also used in approximately one-third of responses to the schizophrenia vignette over the years also illustrates that “depression” is a particularly widespread and possibly now a bit fuzzy term used about mental illness in general, which would partially explain the higher share of correct labelling in responding to the depression vignette. The increased occurrence of responses containing both correct and incorrect terminology for both vignettes since 1990 is consistent with a transition of psychiatric terminology from professional jargon to common usage but also reveals uncertainty among respondents regarding the appropriate use of such terminology. The normalization of depression is also reflected in the increase in trivializing comments like “They should just get some rest or take a holiday”. Together with a decrease in clearly stigmatizing language, this could be interpreted as a decrease in stigmatizing attitudes. However, trivialization of a severe disorder like major depression devalues the illness experiences of those affected. Our study also included quantitative measures of stigma, like the desire for social distance, the most widely used quantitative measure for discriminatory attitudes [39–41]. These results have been published elsewhere [22]. Emotional reactions got somewhat more compassionate and less uncomfortable and annoyed between 1990 and 2020 [22], and between 2011 and 2020, continuum beliefs concerning the depression vignette increased [42]. A study in the US, monitoring time trends in mental illness stigma from 1996 to 2018, found that in the most recent survey, the depression vignette elicited reactions similar to a vignette describing a “troubled person” without any psychiatric illness [43], clearly illustrating the normalization of depressive symptoms.

For the schizophrenia vignette, such a normalization cannot be found. A fifth of respondents consistently made stigmatizing statements, with a marked peak in 2001. The pronounced use of derogatory language in both schizophrenia and depression vignettes in 2001 reflects the dominance of a biogenetic model for mental illness aetiology, notably prevalent during the 1990s, termed the “Decade of the Brain.” [13, 44–47]. The quantitative results of the same surveys show an increase in the desire for social distance towards someone with schizophrenia and an increase in negative emotions like fear or feeling uncomfortable [22]. Continuum beliefs decreased between 2011 and 2020 [23, 48]. In the present study, respondents more frequently mislabelled or entirely overlooked schizophrenia, mistakenly identifying it as depression [22, 42, 49, 50]. Hence, taken together, our findings do not support the conclusion that increased illness recognition indicates a reduction in stigmatizing attitudes among the general population.

Overall, our study rather substantiates that mental health literacy in the German population regarding schizophrenia and major depression has markedly improved, yet stigmatizing or trivializing terminology has not decreased. Therefore, in addition to efforts to educate the public and raise awareness of mental health issues, dedicated efforts to reduce the stigma, particularly of severe mental disorders, are still urgently needed.

## Supplementary Information

Below is the link to the electronic supplementary material.

Supplementary file1 (DOCX 58 KB)

## Data Availability

The data that support the findings of this study are available on request from the senior author GS.
