# Supplementary material for: Changes in the ability to correctly identify schizophrenia and depression: results from general population surveys in Germany over 30 years
Source: Soc Psychiatry Psychiatr Epidemiol. 2024 Apr 7;59(10):1793–801. doi: 10.1007/s00127-024-02660-y (PMC11481663; doi:10.1007/s00127-024-02660-y)
Supplement: Supplementary file 1 — Supplementary file1 (DOCX 58 KB) [file 127_2024_2660_MOESM1_ESM.docx]

Changes in the ability to correctly identify schizophrenia and depression – results from general population surveys in Germany over 30 years.

Elise Grohmann, Amani Al-Addous, Christian Sander, Ezgi Dogan-Sander, Eva Baumann, Matthias C. Angermeyer, Georg Schomerus

**Supplementary Material**

**Explanation of the three-stage random method**

First, the region was selected based on the community structure of Germany, the intercommunal area structure and the electronic regional structure created for navigation system classifications. In the subsequent second selection stage, individual target households were identified using the random route method. The target person was determined within the household using the “Kish selection grid”.

**Case-Vignettes (male version)**

*Schizophrenia*

Please imagine that you find out the following about an acquaintance with whom you are occasionally doing something in your free time:

Within the past six months, your acquaintance appears to have changed. More and more, he retreated from his friends and colleagues, up to the point of avoiding them. If someone managed to involve him in a conversation, he would address only one single topic: whether some people had the natural gift of reading other people’s thoughts. This question became his sole concern. In contrast with his previous habits, he stopped taking care of his appearance and looked increasingly untidy. At work, he seemed absent-minded and frequently made mistakes. Consequently, he has already been summoned by his boss.

Finally, your acquaintance stayed away from work for an entire week without an excuse. Upon his return, he seemed anxious and hounded. He reports that he is now certain, that people cannot only read other people’s thoughts but that they also directly influence them. He was however unsure who would steer his thoughts. He also said that, when thinking, he was continually interrupted. Frequently, he would even hear those people talk to him, and they would give him instructions. Sometimes, they would also talk to each other and make fun of whatever he was doing at the time. The situation was particularly bad at his flat, he claimed. At home, he would really feel threatened and would be terribly scared. Hence, he had not spent the night at his place for the past week, but rather he had hidden in hotel rooms and hardly dared to go out.

*Major depressive Disorder*

Please imagine that you learn the following about an acquaintance with whom you have occasionally done something in your free time for several years:

Within the last two months, your acquaintance has changed a lot in his character. In contrast to before, he is depressed and sad, without being able to give a concrete reason for this. He seems serious-worried. There is nothing he can laugh about anymore. He hardly talks anymore and when he says something, he speaks in a low voice about his worries about the future. Your acquaintance feels useless and has the impression that he is doing everything wrong. Attempts to cheer him up are unsuccessful. He no longer feels like doing anything, nothing interests him. He complains about waking up in the middle of the night and not being able to go back to sleep. In the morning, he already feels tired and without energy. He says he has trouble concentrating on his work. Unlike before, he takes a long time to do everything. He can hardly manage his workload. He has already been summoned to his superior's office for this reason.

**Table S1 Number of survey participants for vignettes of schizophrenia or depression from 1990 to 2020**

|  |  | schizophrenia (*n*) | | depression (*n*) | |
| --- | --- | --- | --- | --- | --- |
|  | Vignette gender | male (*n_m_*) | female (*n_f_*) | male (*n_m_*) | female (*n_f_*) |
| 1990/93 |  | 2115 | | 1492 | |
|  |  | 1573 (74,4%) | 542 (25,6%) | 1004 (67,3%) | 488 (32,7%) |
| 2001 |  | 2481 | | 2544 | |
|  |  | 2481 | - | 2544 | - |
| 2011 |  | 1235 | | 1220 | |
|  |  | 618 (50%) | 617 (50%) | 614 (50,3%) | 606 (49,7%) |
| 2020 |  | 1512 | | 1530 | |
|  |  | 753 (49,8%) | 759 (50,2%) | 768 (50,2%) | 762 (49,8%) |

Total number of respondents (*n, sample size*) and allocation of male and female vignettes (*n_m_, n_f_*) for the survey waves 1990/93, 2001, 2011 and 2020, collected in West and East Germany.

**Table S2 Main categories in the response coding system used in all four survey waves.**

| *Category* | *Description* | *Examples* |
| --- | --- | --- |
| medical language | all answers that contained a technical term | schizophrenia, psychotic, neurotic disorder, dementia, autism, phobia, borderline |
| everyday language | everyday terms to describe the problem in a medical context | split personality, mentally damaged, something with the nerves, morbid, mental strain, crazy |
| physical illnesses or problems | cause of the problem described in physical symptoms | heart attack, menopause, unhealthy lifestyle |
| addiction to alcohol | cause for the problem described concerning alcohol | alcoholic, drinker, takes refuge in alcohol |
| addiction to hard drugs | cause of the problem described concerning hard drugs | drug abuse, withdrawal (problems), addiction to pills |
| personality problems | The cause of the problem described is related to the individual's personality structure or personality traits. | Lack of self-confidence, unable to cope with life, oversensitive, unstable individual, self-pitying, malingerer, identity crisis, selfish person, stupid, arrogant |
| crises in various areas of life | causes of the problem described related to problems in personal or work environment, interpretation as a life crisis | problems in relationships, traumatic experiences, no friends, work overload, financial worries, stress, exhausted, disoriented |
| subcultural influences and attachments | causes of the problem described related to cultural influences or attachments | Scientology's church, esotericism, supernatural forces, in contact with spirits, out of touch with reality, bad company – misguided, political issues, influenced by the internet, dropouts, damaged by society |
| impact on third parties/the interviewee | impact on third parties or a connection to the interviewee | we all feel like that sometimes, and that annoys me, I am sorry for him/her. Poses a danger to his/her environment, is a burden for others |
| case fixation – Symptoms of schizophrenia | formulations and words that were used in the respective vignette. | Believes in mind control, hears people talk, stays away from work, withdraws, is afraid |
| case fixation – Symptoms of major depression |  | is sad, feels useless, worries, sleep problems, lack of concentration, unable to work |
| special codes | recommendations for action, unclear / no information | should seek help, should see a psychiatrist, should relax, should pull oneself together, do not know |
| others | answers that do not match the asked question | excellent |

Detailed explanations and exemplars for the main categories utilized in the coding system were applied to analyse responses from all four survey waves.

**Table S3 Groups of categories formed according to the thematic research question.**

|  |  | *Description* | *formed from sub-categories within the following main categories* |
| --- | --- | --- | --- |
| use of medical language (1) | correct diagnosis (1a) | literal mentions of medically correct expressions | medical language |
|  | incorrect diagnosis (1b) | accepted medical terminology, which did not match the respective vignette |  |
|  | correct and incorrect diagnoses (1c) | Cases in which study participants concurrently offered both correct and incorrect diagnoses within a singular response. |  |
|  | Burnout for depression (1d) | For the depression vignette, 'burnout' was considered as a special case. |  |
| Derogatory labels (2) | clearly derogatory labels (2a) | objectively defamatory statements such as “stupid”, “crazy”, “lazy” | everyday language; personality problems; crises in various areas of life; subcultural influences and attachments; impact on third parties/the interviewee |
|  | potential derogation or trivialization (2b) | statements, which could have trivializing and defamatory components such as “should go on holiday”, “relax”, “just sleep in” or “boredom”. | Everyday language; personality problems; crises in various areas of life; subcultural influences and attachments; impact on third parties/the interviewee |

**Table S4 Frequency distribution of thematic groups of categories for a vignette of schizophrenia and major depressive disorder between 1990/93 and 2020**

| *Thematic category group* | *schizophrenia* | | | |  | *depression* | | | |
| --- | --- | --- | --- | --- | --- | --- | --- | --- | --- |
|  | 1990/93  (*n* 2115) | 2001  (*n* 2481) | 2011  (*n* 1235) | 2020  (*n* 1512) |  | 1990/93  (*n* 1492) | 2001  (*n* 2544) | 2011  (*n* 1220) | 2020  (*n* 1530) |
| use of medical language *(1)* | 568 (26.9) | 813 (32.8) | 550 (44.5) | 704 (46.6) |  | 434 (29.1) | 1016 (39.9) | 583 (47.8) | 847 (55.4) |
| correct diagnosis *(1a)* | 371 (17.5) | 555 (22.4) | 377 (30.5) | 519 (34.3) |  | 396 (26.5) | 955 (37.5) | 472 (38.7) | 709 (46.3) |
| incorrect diagnosis *(1b)* | 228 (10.8) | 300 (12.1) | 201 (16.3) | 265 (17.5) |  | 42 (2.8) | 71 (2.8) | 150 (12.3) | 227 (14.8) |
| correct and incorrect diagnoses *(1c)* | 31 (1.5) | 42 (1.7) | 28 (2.3) | 80 (5.3) |  | 4 (0.3) | 10 (0.4) | 39 (3.2) | 89 (5.8) |
| burnout to describe depression *(1d)* |  |  |  |  |  | - | 9 (0.4) | 124 (10.2) | 181 (11.8) |
| depression for schizophrenia | 189 (8.9) | 231 (9.3) | 155 (12.6) | 181 (12.0) |  |  |  |  |  |
| clearly derogatory labels *(2a)* | 410 (19.4) | 676 (27.2) | 219 (17.7) | 272 (18.0) |  | 159 (10.7) | 389 (15.3) | 113 (9.3) | 117 (7.6) |
| potentially derogatory or trivializing labels *(2b)* | 32 (1.5) | 79 (3.2) | 39 (3.2) | 55 (3.6) |  | 6 (0.4) | 48 (1.9) | 7 (0.6) | 80 (5.2) |

Observed numbers of respondents, *n_categories_* (%) acquired in 1990/93, 2001, 2011 and 2020 in West and East Germany using male and female character vignettes of depression or schizophrenia.

**Table S5a Predictors of use of medical language, correct diagnosis, and incorrect diagnosis for schizophrenia in 1990/93 to 2020.**

| Category | Predictor | Predictor estimates | | Predictor evaluation | | Model evaluation | |
| --- | --- | --- | --- | --- | --- | --- | --- |
|  |  | B | Odds ratio Exp(B)  [95% CI] | Wald (*df*=1) | *p* | Omnibus test of model coefficients, χ² | Nagelkerkes R^2^ |
| Use of medical language (yes vs. no) | Time (0=1990/93, 1=2020) | 0.828 | 2.288 [1.959 – 2.673] | 108.776 | <.001*** |  |  |
|  | Education | 0.509 | 1.663 [1.384 – 1.998] | 29.407 | <.001*** |  |  |
|  | Time*Education | -0.062 | 0.940 [0.883 – 1.001] | 3.739 | 0.053 |  |  |
|  | Gender of Vignette | 0.264 | 1.303 [0.956 – 1.774] | 2.813 | 0.093 |  |  |
|  | Time*Gender of Vignette | -0.036 | 0.964 [0.871 – 1.068] | 0.484 | 0.487 |  |  |
|  | Gender of respondent | 0.108 | 1.114 [0.965 – 1.286] | 2.158 | 0.142 |  |  |
|  | Age of respondent (years) | -0.007 | 0.993 [0.988 – 0.997] | 11.048 | 0.001** | 240.623*** | 0.090 |
| correct diagnosis (yes vs. no) | Time (0=1990/93, 1=2020) | 0.848 | 2.334 [1.962 – 2.777] | 91.344 | <.001*** |  |  |
|  | Education | 0.435 | 1.545 [1.251 – 1.906] | 16.397 | 0.001** |  |  |
|  | Time*Education | -0.036 | 0.965 [0.900 – 1.034] | 1.021 | 0.312 |  |  |
|  | Gender of Vignette | -0.048 | 0.953 [0.662 – 1.373] | 0.066 | 0.797 |  |  |
|  | Time*Gender of Vignette | 0.050 | 1.051 [0.937 – 1.179] | 0.725 | 0.395 |  |  |
|  | Gender of respondent | 0.127 | 1.135 [0.968 – 1.331] | 2.429 | 0.119 |  |  |
|  | Age of respondent (years) | -0.008 | 0.992 [0.987 – 0.997] | 10.231 | 0.001** | 202.817*** | 0.083 |
| incorrect diagnosis (yes vs. no) | Time (0=1990/93, 1=2020) | 0.585 | 1.796 [1.454 – 2.218] | 29.559 | <.001*** |  |  |
|  | Education | 0.489 | 1.631 [1.267 - 2.100] | 14.375 | <.001*** |  |  |
|  | Time*Education | -0.106 | 0.899 [0.827 – 0.978] | 6.127 | 0.013* |  |  |
|  | Gender of Vignette | 0.480 | 1.615 [1.062 – 2.456] | 5.033 | 0.025* |  |  |
|  | Time*Gender of Vignette | -0.112 | 0.894 [0.781 – 1.024] | 2.603 | 0.107 |  |  |
|  | Gender of respondent | 0.053 | 1.055 [0.868 – 1.281] | 0.286 | 0.593 |  |  |
|  | Age of respondent (years) | -0.004 | 0.996 [0.990 – 1.002] | 1.423 | 0.233 | 58.944*** | 0.030 |

Results of binary logistic regression analyses with time as the primary predictor, where time represents the effect of time on the manifestation of the following categories: use of medical language and correct and incorrect diagnoses. Gender, age, and education level of the respondents, as well as contact with individuals with mental health disorders and the gender of the vignette, were controlled as potential confounding factors; education, contact, and vignette gender were also considered as potential moderators of the effects of time (interaction terms). * *p* < 0.05, ** *p* < 0.01, *** *p* < 0.001.

**Table S5b Predictors of use of medical language, correct diagnosis, and incorrect diagnosis for depression in 1990/93 to 2020.**

| Category | Predictor | Predictor estimates | | Predictor evaluation | | | Model evaluation | | |  |
| --- | --- | --- | --- | --- | --- | --- | --- | --- | --- | --- |
|  |  | B | Odds ratio Exp(B) [95% CI] | | Wald (*df*=1) | *p* | | Omnibus test of model coefficients, χ² | Nagelkerkes R^2^ | |
| Use of medical language (yes vs. no) | Time (0=1990/93, 1=2020) | 1.088 | 2.969 [2.517 – 3.501] | | 167.406 | <.001*** | |  |  | |
|  | Education | 0.368 | 1.445 [1.177 – 1.773] | | 12.392 | <.001*** | |  |  | |
|  | Time*Education | -0.018 | 0.982 [0.919 – 1.050] | | 0.274 | 0.601 | |  |  | |
|  | Gender of Vignette | 0.001 | 1.001 [0.716 – 1.399] | | 0.000 | 0.996 | |  |  | |
|  | Time*Gender of Vignette | -0.027 | 0.974 [0.875 – 1.083] | | 0.241 | 0.623 | |  |  | |
|  | Gender of respondent | 0.209 | 1.233 [1.057 – 1.438] | | 7.090 | 0.008** | |  |  | |
|  | Age of respondent (years) | -0.005 | 0.995 [0.991 - 1.000] | | 4.020 | 0.045* | | 280.05*** | 0.121 | |
| correct diagnosis (yes vs. no) | Time (0=1990/93, 1=2020) | 0.855 | 2.352 [1.988 – 2.781] | | 99.786 | <.001*** | |  |  | |
|  | Education | 0.438 | 1.550 [1.256 – 1.912] | | 16.746 | <.001*** | |  |  | |
|  | Time*Education | -0.028 | 0.973 [0.909 – 1.041] | | 0.647 | 0.421 | |  |  | |
|  | Gender of Vignette | 0.093 | 1.098 [0.778 – 1.548] | | 0.282 | 0.595 | |  |  | |
|  | Time*Gender of Vignette | -0.060 | 0.941 [0.845 – 1.049] | | 1.187 | 0.276 | |  |  | |
|  | Gender of respondent | 0.225 | 1.252 [1.071 – 1.464] | | 7.986 | 0.005** | |  |  | |
|  | Age of respondent (years) | -0.004 | 0.996 [0.991 – 1.001] | | 2.710 | 0.100 | | 202.677*** | 0.090 | |
| incorrect diagnosis (yes vs. no) | Time (0=1990/93, 1=2020) | 1.782 | 5.943 [4.122 – 8.567] | | 91.209 | <.001*** | |  |  | |
|  | Education | -0.371 | 0.690 [0.383 – 1.243] | | 1.529 | 0.216 | |  |  | |
|  | Time*Education | 0.092 | 1.096 [0.936 – 1.284] | | 1.287 | 0.257 | |  |  | |
|  | Gender of Vignette | -0.882 | 0.414 [0.152 – 1.131] | | 2.960 | 0.085 | |  |  | |
|  | Time*Gender of Vignette | 0.283 | 1.328 [1.016 – 1.735] | | 4.309 | 0.038* | |  |  | |
|  | Gender of respondent | -0.035 | 0.966 [0.745 – 1.253] | | 0.069 | 0.793 | |  |  | |
|  | Age of respondent (years) | -0.008 | 0.992 [0.985 - 1.000] | | 3.659 | 0.056 | | 156.15*** | 0.113 | |

Results of binary logistic regression analyses with time as the primary predictor, where time represents the effect of time on the manifestation of the following categories: use of medical language and correct and incorrect diagnoses. Gender, age, and education level of the respondents, as well as contact with individuals with mental health disorders and the gender of the vignette, were controlled as potential confounding factors; education, contact, and vignette gender were also considered as potential moderators of the effects of time (interaction terms). * *p* < 0.05, ** *p* < 0.01, *** *p* < 0.001.

**Table S6 Distribution of categories for spontaneous responses to the vignette of schizophrenia and major depressive disorder in West (1990) and East Germany (1993).**

|  | *West part of Germany* | *East part of Germany* |
| --- | --- | --- |
| *schizophrenia* | (*n* 1053) | (*n* 1062) |
| use of medical language | 301 (28.6) | 267 (25.1) |
| correct diagnosis | 191 (18.1) | 180 (16.9) |
| incorrect diagnosis | 124 (11.8) | 104 (9.8) |
| correct and incorrect terms | 14 (1.3) | 17 (1.6) |
| clearly derogatory labels | 183 (17.4) | 221 (21.4) |
| potentially derogatory or stigmatizing labels | 15 (1.4) | 17 (1.6) |
|  |  |  |
| *depression* | (*n* 991) | (N 501) |
| use of medical language | 289 (29.2) | 145 (28.9) |
| correct diagnosis | 263 (26.5) | 133 (26.5) |
| incorrect diagnosis | 26 (2.6) | 16 (3.2) |
| correct and incorrect terms | 0 (0.0) | 4 (0.8) |
| clearly derogatory labels | 91 (9.2) | 68 (13.6) |
| potentially derogatory or stigmatizing labels | 4 (0.4) | 2 (0.4) |

Observed percentage distributions of respondents, *n_categories_* (%) were obtained in West Germany in 1990 using male and female vignettes, and in East Germany in 1993 using the male vignette, for schizophrenia and depression.

**Table S7 Use of derogatory language towards individuals with mental illness by Vignette Gender, 1990, 2011, and 2020**

|  |  | Vignette Gender | |
| --- | --- | --- | --- |
|  |  | male | female |
| *schizophrenia* | 1990 | 93 (18.2) | 90 (16.6) |
|  | 2011 | 120 (19.4) | 99 (16) |
|  | 2020 | 139 (18.5) | 133 (17.5 |
|  |  |  |  |
| *depression* | 1990 | 48 (9.5) | 43 (8.8) |
|  | 2011 | 65 (10.6) | 48 (7.9) |
|  | 2020 | 60 (7.8) | 57 (7.5) |

Observed frequency distributions for the use of derogatory language *n_derogatroy language_* (%) based on the vignette gender for the survey waves of 1990, 2011, and 2020.
